# Did Primary Healthcare Patients in Riyadh Experience Their First Migraine Episodes After a Stressful Event, and What Triggers and Relievers Do They Commonly Report?

**DOI:** 10.7759/cureus.74712

**Published:** 2024-11-28

**Authors:** Saleh S Alsuwayt, Yasser S Aljaied, Amani A Ahmed, Fatimah A Al hussain, Hawra M Alawami, Zahra B Alamer, Deema A Aljasser, Maria A Dirani, Waad A Alshanqiti, Hissah M Bin Kanan, Shaimaa T Jamous, Nawal A Alabrazi

**Affiliations:** 1 Family Medicine, King Saud Medical City, Riyadh, SAU; 2 Family Medicine, Dar Al Uloom University, Riyadh, SAU

**Keywords:** migraine, migraine relievers, migraine triggers, migraine with aura, migraine without aura, primary healthcare, riyadh, saudi arabia

## Abstract

Objective: This study aimed to investigate whether the first onset of migraine episodes among primary healthcare patients in Riyadh, Saudi Arabia, is preceded by a highly stressful event, as well as to identify common potential triggers and relievers reported by these patients.

Background: Migraine is a prevalent and debilitating neurological disorder. The pathophysiology of migraine involves complex interactions between genetic, neurological, and environmental factors, including the trigeminovascular system and neuropeptides such as calcitonin gene-related peptide (CGRP).

Methodology: A cross-sectional study was conducted in 2024 involving 271 patients. Data were collected using a Google Forms questionnaire, and analysis was performed using IBM Corp. Released 2023. IBM SPSS Statistics for Windows, Version 29.0.2.0 Armonk, NY: IBM Corp, employing both descriptive and inferential statistics.

Results: A total of 271 participants were included, with a predominance of females (82.3%). The majority (68.3%) reported experiencing a stressful event before their first migraine attack, particularly among the 18-25 age group. The most frequently reported triggers included sleep disturbances (70, 25.8%), emotional stress (53, 19.5%), and hormonal changes in women during the menstrual cycle (28, 10.3%). Relief strategies include falling asleep (101, 37.2%) and food intake (85, 31.3%). A significant association was found between age and the type of migraine experienced, as well as the frequency of attacks.

Conclusions: The findings indicate a notable relationship between stressful events and the onset of migraines, emphasizing the need for targeted management strategies in primary healthcare settings. Understanding the common triggers and effective relief methods can help improve the quality of care for migraine patients in Saudi Arabia.

## Introduction

Migraine is a complex and debilitating neurological disorder that affects an estimated 1 adult in every 7 in the world [1], making it one of the most prevalent neurological conditions globally. A 2023 systematic review and meta-analysis [2] conducted in Saudi Arabia analyzed thirty-six studies with a total of 55,061 participants. The findings revealed a pooled proportion of migraine prevalence in Saudi Arabia of 0.225617, with a 95% confidence interval of 0.172749 to 0.28326, indicating a significant prevalence of migraines in the country. Migraine is characterized by recurrent episodes of throbbing headaches, often accompanied by a range of other disabling symptoms, including nausea, vomiting, sensitivity to light and sound, and visual or auditory disturbances called aura.

The pathophysiology of migraine is not yet fully understood, but it is believed to involve a complex interplay of genetic, neurological, and environmental factors. Current evidence suggests that a primary neuronal dysfunction leads to a sequence of changes intracranially and extracranially that account for migraine, including the four phases: first phase prodrome symptoms, second phase aura, third phase headache, and fourth phase postdrome [3,4]. The vascular theory of migraine, which proposed that migraine headaches were caused by the dilation of blood vessels and that the aura of migraine was due to vasoconstriction, is no longer regarded as a valid explanation for the pathophysiology of migraine [5-7]. Vasodilatation, if it occurs at all during spontaneous migraine attacks [7], is probably an epiphenomenon resulting from instability in the central neurovascular control mechanism [8].

The migraine aura and the accompanying headache have been found to be linked to the neurophysiological process known as cortical spreading depression of Leão [5,9,10]. This evidence supports the idea that there is a causal association between the aura and the subsequent headache experienced during a migraine attack [5,9,10]. The pathophysiology of migraine is believed to involve the activation of the trigeminovascular system. This system is composed of small, pseudounipolar sensory neurons that originate from the trigeminal ganglion and the upper cervical dorsal roots. The involvement of this trigeminovascular system is central to the underlying mechanisms that contribute to the development of migraine [11]. Sensitization is a process in which neurons become increasingly responsive to both nociceptive (pain-related) and non-nociceptive stimuli, which involves a decrease in response thresholds, an increase in response magnitude, an expansion of receptive fields, and the development of spontaneous neuronal activity. Essentially, the neurons involved become more sensitive and reactive to various stimuli. Sensitization is likely responsible for many of the clinical symptoms of migraine, including the throbbing quality of the pain, the worsening of pain with coughing, bending, or sudden head movements (as is often observed during the postdrome), hyperalgesia (increased sensitivity to painful stimuli), and allodynia (pain produced by normally non-noxious stimulation) [12-14]. Although activation of serotonin receptors is known to be important in the acute treatment of migraine, the role of serotonin in the generation of migraine remains unclear. Some researchers have proposed that serotonin, released from serotonergic nuclei in the brainstem, may play a part in the pathogenesis of migraine. This potential involvement could be mediated through serotonin's direct effects on the cranial vasculature, its role in central pain control pathways, or its influence on cerebral cortical projections originating from the brainstem serotonergic nuclei. However, the exact mechanisms by which serotonin may contribute to the underlying pathophysiology of migraine are still not fully understood [15,16]. The calcitonin gene-related peptide has a key role in migraine pathophysiology [17]. The neuropeptide calcitonin gene-related peptide (CGRP) appears to play a key role in the transmission of pain signals from the trigeminovascular system to the central nervous system. CGRP also seems to mediate the vasodilatory component of neurogenic inflammation [17]. Stimulation of the trigeminal ganglion leads to the release of CGRP, and the infusion of CGRP has been shown to induce migraine attacks in individuals with a history of migraine [18]. These findings suggest that CGRP is a critical mediator in the pathophysiology of migraine, linking the activation of the trigeminovascular system to the subsequent development of migraine pain and associated symptoms. Surprisingly, migraine with aura has been linked to the presence of right-to-left cardiac shunts, most often in the form of a patent foramen ovale (PFO), and less frequently with atrial septal defects (ASDs). This suggests a potential relationship between the existence of these cardiac shunt anomalies, which allow blood to flow from the right side of the heart to the left side without passing through the lungs, and the development of migraine attacks accompanied by aura [19-21]. The role of inheritance in migraine has long been recognized. One early study found migraineurs had a 3-fold higher risk in relatives compared to non-migraine controls [22]. Migraines are more prevalent among women, with epidemiological studies indicating 17% of females and 6% of males [23]. The higher incidence among women is thought to be related to hormonal factors, as fluctuations in estrogen levels have been linked to the onset and exacerbation of migraine symptoms. Additionally, migraine has been found to be comorbid with other neurological, psychiatric, and cardiovascular conditions, further complicating its management and highlighting the need for a comprehensive, multidisciplinary approach to patient care. The burden of migraine on individuals and society is significant. Migraine attacks can be severely debilitating, causing missed work or school days, reduced productivity, and impaired social and daily functioning. The economic impact of migraine is also substantial; the total indirect cost associated with migraine in the United States has been estimated at $19.3 billion (inflated to 2019 US$) [24], 81% of which is attributable to absenteeism (full days of productive workforce loss) [25]. According to an evidence-based review [26], several environmental and dietary factors have been identified as potential triggers for migraine attacks. The review concluded [26] that stress, menstrual cycles, visual stimuli, weather changes, nitrates, fasting, and consumption of wine were likely migraine-trigger factors. Additionally, sleep disturbances and the artificial sweetener aspartame were considered possible migraine triggers [26]. In a retrospective study involving 1,750 patients with migraine, around 75% of the participants reported experiencing at least one trigger that precipitated their acute migraine attacks [27]. The triggers were identified in the following order, from most to least frequent: Emotional stress (80%), hormones in females (65%), fasting (57%), hot weather (53%), sleep disturbances (50%), odors (44%), neck pain (38%), bright lights (38%), alcohol intake (38%), cigarette smoking (36%), sleeping late (32%), environmental heat (30%), spicy food (27%), aerobic exercise (22%), and sexual activity (5%).

## Materials and methods

This cross-sectional study was conducted in 2024 at a primary healthcare clinic in Riyadh, Saudi Arabia. Participants in the study were required to be between the ages of 18 and 75 years. They must have screened positive on the ID Migraine Screening Tool, achieving a score of two or higher, and must have a known diagnosis of migraine.

Conversely, individuals were excluded from the study if they were younger than 18 or older than 75 years, if they were pregnant, or if they had mental or physical disabilities. Additionally, those with any other comorbidities were also excluded. Lastly, patients who were not diagnosed with migraine and who scored below two on the ID Migraine Screening Tool were not eligible to participate in the study.

In this study, a minimum target sample size of 271 participants was established to achieve a confidence level of 95%, a margin of error of 5%, and a population proportion of 22.56%. This determination was based on a systematic review conducted in 2023 in Saudi Arabia [2]. To minimize selection bias, random sampling was employed, ensuring that each participant from the targeted population had an equal chance of being included in the study.

The research was conducted at the primary healthcare facilities of the Riyadh First, Second, and Third Health Clusters. Data collection took place over a period spanning from July 1, 2024, to October 15, 2024.

For the purpose of screening migraines, the ID Migraine Screening Tool was utilized. This tool consists of three key questions aimed at assessing the presence of migraine symptoms. The questions asked participants whether, during the past three months, they had experienced the following:

Did you feel nauseated or sick to your stomach?

Did light bother you a lot more than when you didn’t have headaches?

Did your headaches limit your ability to work, study, or do what you needed to do?

If participants answered "yes" to at least two out of these three questions, they had an 81% chance of having a migraine.

Data were collected directly from participants who consented to take part in the research. The structured questionnaire included the ID Migraine Screening Tool alongside demographic questions and other relevant health information. Participants were approached in various primary healthcare settings, and informed consent was obtained prior to their participation.

The questionnaire was developed based on existing literature regarding migraine.

A multi-step process was implemented to ensure the validity and reliability of the questionnaire. This included pilot testing with a small group of 30 participants to assess the clarity and relevance of the questions, followed by adjustments based on their feedback. Additionally, the questionnaire underwent validation through a review by experts in neurology, family medicine, and epidemiology.

Statistical analysis for this cross-sectional study was conducted using IBM Corp. Released 2023. IBM SPSS Statistics for Windows, Version 29.0.2.0 Armonk, NY: IBM Corp. Descriptive statistics were utilized to summarize participant demographics and clinical history, while Chi-Square tests were employed to examine associations between categorical variables, which include demographic data, clinical characteristics of participants, common migraine triggers reported by participants, common migraine relief methods reported by participants, stressful events occurring before the first migraine attack, migraine severity, and the type of migraine experienced.

## Results

A total of 271 participants were included in this study. The majority were female, making up 82.3% (223 individuals), while males accounted for 17.7% (48 individuals). Most participants were Saudi nationals, representing 75.6% (205 individuals), with 24.4% (66 individuals) non-Saudi.

The age distribution revealed that the largest group was individuals aged 31-40 years, comprising 39.9% (108 individuals). This was followed by the 61-70 years group at 29.5% (80 individuals) and those aged 41-50 years at 19.9% (54 individuals). Smaller numbers were seen in the 51-60 years group (5.9%, 16 individuals) and the 18-30 years group (4.8%, 13 individuals). The demographic data reveals a predominantly female population (82.3%), suggesting a higher prevalence of migraines among women. Most participants are Saudi (75.6%), with 39.9% aged 31-40, highlighting significant stressors in this life phase. Additionally, 47.2% are employed, linking work-related stress to migraines. A notable 66.8% hold at least a diploma, which could impact more individuals with education levels beyond high school. The participants represent diverse income levels: 11.4% earn less than 5,000 SAR, 26.9% earn between 5,000-10,000 SAR, 32.8% earn between 10,000-20,000 SAR, and 28.8% earn more than 20,000 SAR, indicating a higher frequency of migraines among those with incomes of 10,000 SAR and above. Finally, with 55.7% being married, social support dynamics are relevant to migraine experiences. More detailed demographic data is presented in Table 1.

**Table 1:** Demographic data.

	Category	Frequency	Percent (%)
Gender	Female	223	82.3
	Male	48	17.7
	Total	271	100.0
Nationality	Saudi	205	75.6
	Non-Saudi	66	24.4
	Total	271	100.0
Age	18-30	13	4.8
	31-40	108	39.9
	41-50	54	19.9
	51-60	16	5.9
	61-70	80	29.5
	Total	271	100.0
Employment Status	Student	33	12.2
	Employee	128	47.2
	Non-employee	90	33.2
	Retired	9	3.3
	Working for personal account	11	4.1
	Total	271	100.0
Education Level	Not educated	1	0.4
	Educated (primary-secondary)	45	16.6
	Education (Diploma-bachelor's)	181	66.8
	Postgraduate (Masters's-Doctorate)	44	16.2
	Total	271	100.0
Monthly Income	Less than 5000 SAR	31	11.4
	5000-10000 SAR	73	26.9
	10000-20000 SAR	89	32.8
	More than 20000 SAR	78	28.8
	Total	271	100.0
Marital Status	Single	93	34.3
	Married	151	55.7
	Divorced	23	8.5
	Widowed	4	1.5
	Total	271	100.0

The clinical characteristics of the 271 participants revealed that a significant majority, 66.1% (179 individuals), reported having received a formal diagnosis of migraines from a healthcare provider. The remaining 33.9% (92 individuals) were included in the study based on positive scores from the ID Migraine screening tool.

Regarding the age of first migraine onset, most patients (45.8%, 124 individuals) experienced their first migraine between the ages of 18 and 25, followed by 33.9% (92 individuals) who reported their first migraine between 25 and 30 years of age.

The frequency of migraine attacks varied considerably among participants: 41.7% (113 individuals) reported experiencing 2 to 4 attacks per month, 29.5% (80 individuals) had 12 or fewer attacks per year, and 21.0% (57 individuals) experienced more than one attack per week. Only 7.7% (21 individuals) reported having just one attack annually.

In terms of severity, the majority of patients described their migraines as moderate (5-6 on a subjective scale of 1-10), accounting for 47.6% (129 individuals). Severe migraines (rated 7-10) were reported by 42.4% (115 individuals), while a smaller group (10.0%, 27 individuals) classified their migraines as mild (1-4).

Interestingly, 43.2% (117 individuals) indicated that the frequency of their migraines increased with age, while 56.8% (154 individuals) did not observe this trend. Family history was assessed, revealing that 42.1% (114 individuals) reported a family history of migraines, 15.5% (42 individuals) noted a family history of neurological disorders, and 30.3% (82 individuals) had a family history of heart disorders.

Additionally, 68.3% (185 individuals) reported experiencing stressful events prior to their first migraine attack, suggesting that stress may be a potential trigger for migraine development. Furthermore, 81.2% (220 individuals) identified specific migraine triggers, with 50.9% (138 individuals) indicating a high likelihood of experiencing a migraine when exposed to these triggers. In contrast, 38.4% (104 individuals) rated the likelihood as medium, while 10.7% (29 individuals) rated it as low. A more detailed description of the clinical characteristics is shown in Table 2.

**Table 2:** Clinical characteristics of participants.

	Category	Frequency	Percent (%)
Formally diagnosed with migraine	Yes	179	66.1
	No	92	33.9
	Total	271	100.0
Age of first ever migraine attack	18-25	124	45.8
	26-30	92	33.9
	31-35	28	10.3
	36-40	15	5.5
	41+	12	4.4
	Total	271	100.0
Last time you had migraine attack	Within last 12 months	19	7.0
	3-6 months ago	34	12.5
	1-2 months ago	47	17.3
	Less than a month ago	171	63.1
	Total	271	100.0
Migraine frequency per year	Once a year	21	7.7
	12 or fewer attacks per year	80	29.5
	2 to 4 attacks per month	113	41.7
	More than 1 attack per week	57	21.0
	Total	271	100.0
Does Frequency of migraine attack increase with age	Yes	117	43.2
	No	154	56.8
	Total	271	100.0
Migraine severity (1-10 scale)	1-4 mild	27	10.0
	5-6 moderate	129	47.6
	7-10 severe	115	42.4
	Total	271	100.0
Family history of migraine	Yes	114	42.1
	No	157	57.9
	Total	271	100.0
Family history of neurological disorders	Yes	42	15.5
	No	229	84.5
	Total	271	100.0
Family history of heart disorders	Yes	82	30.3
	No	189	69.7
	Total	271	100.0
Stressful experiences before first ever migraine attack	Yes	185	68.3
	No	86	31.7
	Total	271	100.0
Specific migraine triggers	Yes	220	81.2
	No	51	18.8
	Total	271	100.0
likelihood of experiencing a migraine attack when exposed to these triggers	High (almost permanent)	138	50.9
	Medium	104	38.4
	Low	29	10.7
	Total	271	100.0

Table 3 shows the various triggers reported by participants associated with their migraine episodes. The most commonly reported trigger is sleep disturbance, noted by 70 participants (25.8%). Emotional stress followed, reported by 53 participants (19.5%). Additionally, neck pain and hormonal changes in women during the menstrual cycle were each reported by 28 participants (10.3%).

**Table 3:** The common migraine triggers reported by participants.

Migraine Triggers	Frequency	Percentage
Sleep disturbance	70	25.8%
Emotional stress	53	19.6%
Neck pain	28	10.3%
Hormonal changes in women during menstrual cycle	28	10.3%
Bright light	26	9.6%
Not eating for longer than usual	21	7.7%
Odors	17	6.3%
Cigarettes smoking	5	1.8%
Hot weather	4	1.5%
Physical exercise	4	1.5%
Jaw biting	4	1.5%
Loud noises	3	1.1%
Alcohol Intake	2	0.7%
Sexual activity	2	0.7%
Exhaustion	2	0.7%
Sinusitis	2	0.7%
Total	271	100%

Table 4 shows the various relief methods used by participants during their migraine episodes. The most common relief method is falling asleep, as noted by 101 participants (37.2%). Food intake followed, reported by 85 participants (31.3%), and practicing physical exercise was reported by 27 participants (9.9%).

**Table 4:** Common migraine relief methods reported by participants.

Relief Method	Frequency	Percentage
Falling asleep	101	37.2%
Food intake	85	31.4%
Practicing physical exercise	27	10.0%
Limiting caffeine intake	19	7.0%
Medication: codeine, acetaminophen, ibuprofen	18	6.6%
Diet (cutting sugar and carbs in meals, cutting dairy)	5	1.8%
Avoid loud noises	3	1.1%
Dim light, darkness	3	1.1%
Cold water shower	2	0.7%
Head massage and neck	2	0.7%
Relaxing	2	0.7%
Avoid stress	2	0.7%
Multivitamin	1	0.4%
Vomiting	1	0.4%
Total	271	100%

Stressful events before the first-ever migraine attack

We examine the relationship between the age at which participants first ever experienced a migraine attack and the occurrence of stressful events preceding these attacks. The findings shed light on the prevalence of stressful experiences prior to the first-ever migraine episode across various age groups.

As illustrated in Table 5 below, a significant majority of participants (68.3%, 185 individuals) reported that their first-ever migraine attack was preceded by a stressful event. The age group most affected was 18-25 years, where 31.0% (84 individuals) indicated that they had not experienced any stressful events before their first-ever migraine. Conversely, participants in older age groups (31-35 years, 36-40 years, and 41+ years) reported lower incidences of stress prior to their first-ever migraine attack. For instance, only 3.7% (10 individuals) in the 41+ age group reported experiencing a stressful event before their first-ever migraine attack.

**Table 5:** Stressful Events Before the First Ever Migraine Attack. Chi-square test

	Stressful Event Preceded First Ever Migraine Attack			p-value
Age first ever experience migraine attacks	Yes	No	Total	
18-25 years	84 (31.0%)	40 (14.8%)	124 (45.8%)	0.534
26-30 years	59 (21.8%)	33 (12.2%)	92 (33.9%)	
31-35 years	21 (7.7%)	7 (2.6%)	28 (10.3%)	
36-40 years	11 (4.1%)	4 (1.5%)	15 (5.5%)	
41+ years	10 (3.7%)	2 (0.7%)	12 (4.4%)	
Total	185 (68.3%)	86 (31.7%)	271 (100%)	

A Chi-square test was conducted to determine if there is a statistically significant association between age at first migraine onset and whether a stressful event preceded the migraine. The p-value for this test is 0.534, indicating no statistically significant association between the age of the first-ever migraine and the occurrence of a stressful event. This suggests that stressful events are not more likely to precede migraine onset in any particular age group.

Migraine severity and type of migraine experienced

We examined the relationship between the type of migraine experienced by participants (without aura and with aura) and the reported severity of their migraine attacks on a subjective scale from 1-10, categorized as mild (1-4), moderate (5-6), or severe (7-10). Understanding this relationship helps determine if the type of migraines is associated with more severe symptoms.

Table 6 below shows that among participants who experience migraines without aura, 22.1% (60 individuals) reported severe migraines, while 28.4% (77 individuals) described their migraines as moderate, and 8.9% (24 individuals) rated their migraines as mild (1-4). In contrast, among participants who have migraines with aura, 20.3% (55 individuals) reported severe migraines, 19.2% (52 individuals) experienced moderate migraines, and only 1.1% (3 individuals) classified their migraines as mild.

**Table 6:** Migraine Severity and Type of Migraine Experienced. Chi-square test

Type of Migraine Experienced	Mild (1-4)	Moderate (5-6)	Severe (7-10)	Total	p-value
Without Aura	24 (8.9%)	77 (28.4%)	60 (22.1%)	161 (59.4%)	0.002
With Aura	3 (1.1%)	52 (19.2%)	55 (20.3%)	110 (40.6%)	
Total	27 (10.0%)	129 (47.6%)	115 (42.4%)	271 (100%)	

A Chi-square test was conducted to determine whether there is a statistically significant association between the type of migraine experienced and the severity of migraine symptoms. The P-value for this test is 0.002, indicating a statistically significant relationship between migraine type and severity. This suggests that participants with migraines without aura are more likely to experience moderate to severe migraines compared to those with migraines with aura, who also report high severity but with slightly different distributions across severity categories.

Association between migraine type and age

This analysis examined the association between age and the type of migraine experienced by participants. The distribution of migraine types was analyzed across various age groups as shown in Table 7.

**Table 7:** Association Between Migraine Type and Age. Pearson Chi-square

Age Group	Migraine without Aura	Migraine With Aura	Total	p-value
18-30 years	13 (8.1%)	0 (0.0%)	13 (4.8%)	0.022
31-40 years	63 (39.1%)	45 (40.9%)	108 (39.9%)	
41-50 years	29 (18.0%)	25 (22.7%)	54 (19.9%)	
51-60 years	7 (4.3%)	9 (8.2%)	16 (5.9%)	
61-70 years	49 (30.4%)	31 (28.2%)	80 (29.5%)	
Total	161 (100.0%)	110 (100.0%)	271 (100.0%)	

The analysis of the association between age and migraine type reveals significant trends, particularly in how migraine prevalence varies across different age groups, as shown by a Pearson Chi-square value of 11.402 and a p-value of 0.022, showing a notable relationship between age and migraine type; specifically, in the 18-30 age group, all 13 participants experienced Migraine without Aura, suggesting a higher prevalence of this type among younger individuals, while the 31-40 age group exhibited a balanced distribution with 39.1% experiencing Migraine without Aura and 40.9% with Migraine with Aura, similar to the 41-50 age group, which also showed near-equilibrium in the prevalence of both migraine types. However, in the 51-60 age group, the prevalence dropped significantly, with only 4.3% experiencing migraine without aura and 8.2% with migraine with aura, but we raise questions about the reliability of these figures in this age group 51-60 due to the smaller sample size in this age group. Conversely, the 61-70 age group saw a resurgence in both types: 30.4% migraine without aura and 28.2% migraine with aura.

Association between migraine frequency and age

The association between age and the frequency of migraine attacks was analyzed to understand how migraine frequency varies across different age groups, as shown in Table 8.

**Table 8:** Association Between Migraine Frequency and Age. Pearson Chi-square

Age Group	Once a Year	12 or Fewer per Year	2-4 Attacks per Month	More than 1 Attack per Week	Total	p-value
18-30 years	7 (33.3%)	3 (3.8%)	3 (2.7%)	0 (0.0%)	13 (4.8%)	0.001
31-40 years	6 (28.6%)	30 (37.5%)	50 (44.2%)	22 (38.6%)	108 (39.9%)	
41-50 years	5 (23.8%)	24 (30.0%)	14 (12.4%)	11 (19.3%)	54 (19.9%)	
51-60 years	0 (0.0%)	3 (3.8%)	11 (9.7%)	2 (3.5%)	16 (5.9%)	
61-70 years	3 (14.3%)	20 (25.0%)	35 (31.0%)	22 (38.6%)	80 (29.5%)	
Total	21(100.0%)	80(100.0%)	113(100.0%)	57(100.0%)	271(100.0%)	

The Pearson Chi-square value was 57.571, with a p-value of less than 0.001, indicating a highly significant association between age and migraine frequency. This suggests that the frequency of migraine attacks is significantly influenced by age.

## Discussion

The primary focus of this research was to investigate whether patients experienced a stressful event prior to their first-ever migraine attack. A significant majority of participants (68.3%, n = 185) reported that their initial migraine attack was preceded by a stressful event. This aligns with research conducted by Almojil et al. (2021) [28], which reported that psychological stress is a prominent trigger for migraines in a similar demographic. In their study, 81.6% of participants identified stress as a contributing factor, highlighting the critical role of emotional well-being in migraine pathophysiology [28].

The age group with the highest proportion of individuals reporting such events was 18-25 years. Conversely, 31.7% (n = 86) of participants did not report a stressful event before their first migraine attack.

Moreover, the predominance of female participants (82.3%) in our study is consistent with the literature, which often cites a higher prevalence of migraines among women due to hormonal fluctuations by Smith et al., 2022 [29]. This demographic similarity underscores the need for targeted interventions that consider gender-specific factors in migraine management.

To explore the relationship between age at first migraine onset and the occurrence of stressful events, a chi-square test was performed. The resulting p-value of 0.534 indicated no statistically significant association between the age of first migraine onset and the presence of a preceding stressful event. This finding suggests that stressful events are not uniformly linked to migraine onset across different age groups.

Interestingly, the most common age group for the onset of the first migraine attack was 18-25 years, comprising 45.8% (n = 124) of participants. As age increased, the incidence of migraine attacks decreased significantly: 33.9% (n = 92) in the 26-30 age group, 10.3% (n = 28) in the 31-35 group, 5.5% (n = 15) in the 36-40 group, and 4.4% (n = 12) in the 41+ age group.

In terms of migraine severity, most participants (47.6%, n = 129) rated their migraine attacks as moderate on a subjective scale of 1-10, where 1-4 is classified as mild, 5-6 as moderate, and 7-10 as severe. Furthermore, a majority of participants reported no family history of neurological or cardiovascular conditions (84.5%, n = 229, and 69.7%, n = 189, respectively). In contrast, 42.1% (n = 114) reported a family history of migraines.

A significant proportion of participants (81.2%, n = 220) identified specific migraine triggers. The most frequently reported trigger was sleep disturbance, noted by 70 participants, followed closely by emotional stress, 53 participants. Hormonal changes during menstruation were reported as triggers by 28 women, while neck pain was also noted as a trigger by 28 participants. The likelihood of experiencing a migraine attack when exposed to these triggers ranged from "almost always" to "occasionally" among 89.3% (n = 242) of participants. This aligns with a 2021 study conducted in Saudi Arabia, which identified sleep deprivation (94%) and stress/anxiety (81.6%) as prevalent triggers [30].

Relief methods were also explored, with the most frequently mentioned strategy being falling asleep, cited by 101 participants. Food intake was reported by 85 participants as another critical strategy. Practicing physical exercise was noted by 27 participants.

We also analyzed the relationship between age and the type of migraine, distinguishing between those with and without aura. The Pearson Chi-square value was 11.402 with a p-value of 0.022, suggesting a statistically significant association between age and migraine type. Specifically, individuals aged 31-40 experienced migraine with aura at the highest rate (40.9%, n = 45).

Furthermore, we examined the association between age and the frequency of migraine attacks. The Pearson Chi-square value was 57.571 with a p-value of less than 0.001, indicating a highly significant relationship. The age groups with the most frequent attacks were 31-40 years (n = 108, 39.9%) and 61-70 years (n = 80, 29.5%).

Limitations

This study has several limitations that warrant consideration: A) Retrospective recall: Many participants struggled to accurately identify the onset of their first migraine attack. This reliance on retrospective recall may introduce inaccuracies and biases that could affect the reliability of our findings. B) Geographic limitation: Data collection was restricted to Riyadh city, potentially limiting the generalizability of our results to other regions within Saudi Arabia. C) Potential selection bias: Participants were selected based on clinically diagnosed migraines and the ID Migraine Screening Tool. This may have resulted in a sample that does not fully represent individuals with varying degrees of migraine severity, including those missed clinically or those who did not score positive on the screening tool. Although a systematic review in 2011 found the ID Migraine tool has a sensitivity of 81% in various clinical settings [31], this limitation may affect the applicability of our findings.

By acknowledging these limitations, we provide context for our results and emphasize the need for further research involving a more diverse sample and improved selection criteria to validate these findings.

## Conclusions

In conclusion, this research highlights the significant relationship between stressful events and the onset of migraine attacks, with 68.3% of participants reporting a stressful event preceding their first migraine. This finding aligns with existing literature, reinforcing the notion that psychological stress plays a critical role in migraine pathophysiology. Notably, the highest incidence of reported stressful events occurred within the 18-25 age group, indicating a potential vulnerability in younger adults that warrants further exploration. The study's demographic profile, characterized by a predominance of female participants, echoes previous research that links gender-specific factors, particularly hormonal fluctuations, to migraine prevalence. This underscores the importance of tailoring interventions to address the unique experiences of different demographic groups. Despite the findings indicating that stressful events are common precursors to migraine onset, our analysis revealed no statistically significant association between the age of initial migraine onset and the occurrence of these events. This suggests that while stress may trigger migraines, its impact does not uniformly influence the age at which individuals experience their first attack. Additionally, the data revealed that sleep disturbances and emotional stress are among the most frequently identified migraine triggers, corroborating findings from other studies. The exploration of relief methods also sheds light on the coping strategies employed by participants, with sleep being the most commonly cited method for alleviating migraine symptoms.

However, the study is not without limitations. The reliance on retrospective recall may introduce biases, and the geographic scope confined to Riyadh limits the generalizability of the findings across diverse populations in Saudi Arabia. Furthermore, potential selection bias due to the criteria for participant inclusion could affect the representativeness of the sample. In summary, while the findings contribute valuable insights into the relationship between stress and migraine onset, they also highlight the need for further research. Future studies should aim for a more extensive and diverse sample to validate and expand upon these findings, ultimately leading to improved understanding and management of migraines in various populations.
